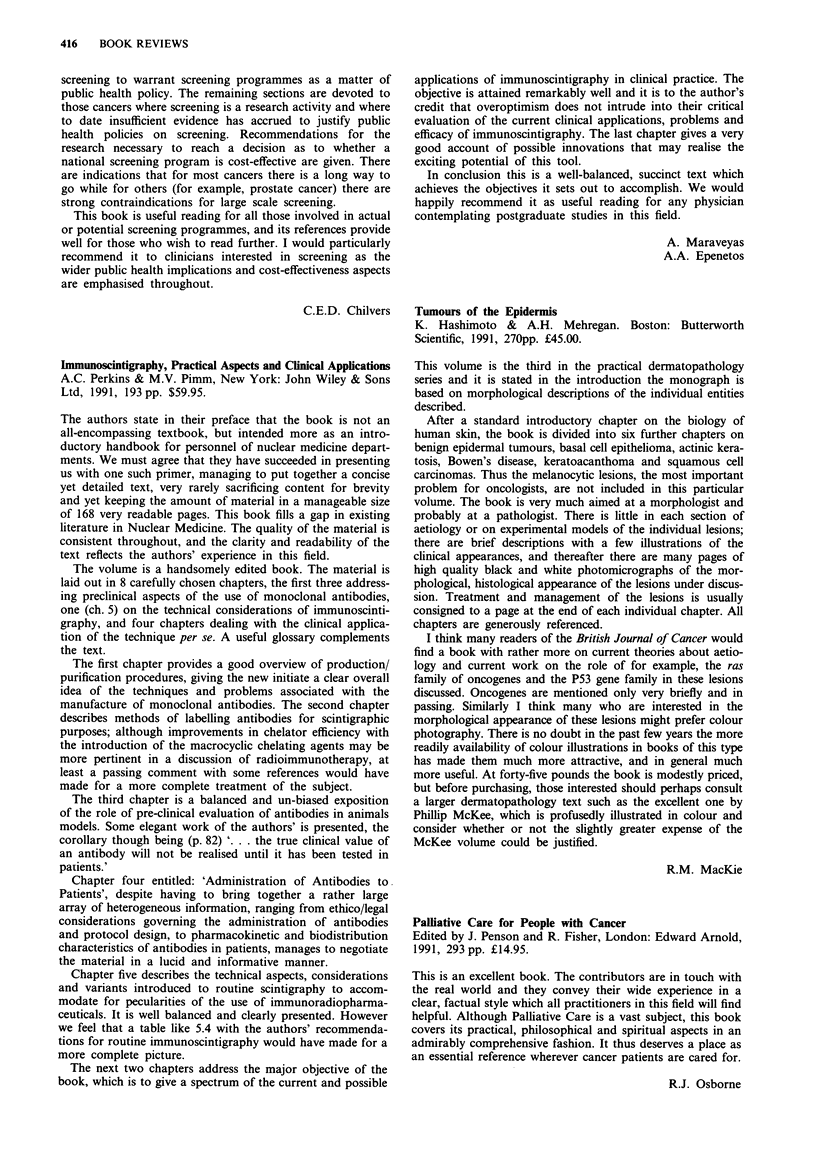# Immunoscintigraphy, Practical Aspects and Clinical Applications

**Published:** 1992-08

**Authors:** A. Maraveyas, A.A. Epenetos


**Immunoscintigraphy, Practical Aspects and Clinical Applications**
A.C. Perkins & M.V. Pimm, New York: John Wiley & Sons Ltd, 1991, 193 pp. $59.95.

The authors state in their preface that the book is not an all-encompassing textbook, but intended more as an introductory handbook for personnel of nuclear medicine departments. We must agree that they have succeeded in presenting us with one such primer, managing to put together a concise yet detailed text, very rarely sacrificing content for brevity and yet keeping the amount of material in a manageable size of 168 very readable pages. This book fills a gap in existing literature in Nuclear Medicine. The quality of the material is consistent throughout, and the clarity and readability of the text reflects the authors' experience in this field.

The volume is a handsomely edited book. The material is laid out in 8 carefully chosen chapters, the first three addressing preclinical aspects of the use of monoclonal antibodies, one (ch. 5) on the technical considerations of immunoscintigraphy, and four chapters dealing with the clinical application of the technique *per se*. A useful glossary complements the text.

The first chapter provides a good overview of production/purification procedures, giving the new initiate a clear overall idea of the techniques and problems associated with the manufacture of monoclonal antibodies. The second chapter describes methods of labelling antibodies for scintigraphic purposes; although improvements in chelator efficiency with the introduction of the macrocyclic chelating agents may be more pertinent in a discussion of radioimmunotherapy, at least a passing comment with some references would have made for a more complete treatment of the subject.

The third chapter is a balanced and un-biased exposition of the role of pre-clinical evaluation of antibodies in animals models. Some elegant work of the authors' is presented, the corollary though being (p. 82) '. . . the true clinical value of an antibody will not be realised until it has been tested in patients.'

Chapter four entitled: 'Administration of Antibodies to Patients', despite having to bring together a rather large array of heterogeneous information, ranging from ethico/legal considerations governing the administration of antibodies and protocol design, to pharmacokinetic and biodistribution characteristics of antibodies in patients, manages to negotiate the material in a lucid and informative manner.

Chapter five describes the technical aspects, considerations and variants introduced to routine scintigraphy to accommodate for pecularities of the use of immunoradiopharmaceuticals. It is well balanced and clearly presented. However we feel that a table like 5.4 with the authors' recommendations for routine immunoscintigraphy would have made for a more complete picture.

The next two chapters address the major objective of the book, which is to give a spectrum of the current and possible applications of immunoscintigraphy in clinical practice. The objective is attained remarkably well and it is to the author's credit that overoptimism does not intrude into their critical evaluation of the current clinical applications, problems and efficacy of immunoscintigraphy. The last chapter gives a very good account of possible innovations that may realise the exciting potential of this tool.

In conclusion this is a well-balanced, succinct text which achieves the objectives it sets out to accomplish. We would happily recommend it as useful reading for any physician contemplating postgraduate studies in this field.

A. Maraveyas
A.A. Epenetos